# Emerging PCR-Based Techniques to Study HIV-1 Reservoir Persistence

**DOI:** 10.3390/v12020149

**Published:** 2020-01-28

**Authors:** Laurens Lambrechts, Basiel Cole, Sofie Rutsaert, Wim Trypsteen, Linos Vandekerckhove

**Affiliations:** 1 HIV Cure Research Center, Department of Internal Medicine and Pediatrics, Ghent University, 9000 Ghent, Belgium; laurens.lambrechts@ugent.be (L.L.); Basiel.Cole@UGent.be (B.C.); Sofie.Rutsaert@UGent.be (S.R.); Wim.Trypsteen@UGent.be (W.T.); 2 BioBix, Department of Data Analysis and Mathematical Modelling, Faculty of Bioscience Engineering, Ghent University, 9000 Ghent, Belgium

**Keywords:** HIV-1, HIV-1 reservoir, PCR, sequencing, NGS, replication-competent, integration site, HIV-1 genome

## Abstract

While current antiretroviral therapies are able to halt HIV-1 progression, they are not curative, as an interruption of treatment usually leads to viral rebound. The persistence of this stable HIV-1 latent reservoir forms the major barrier in HIV-1 cure research. The need for a better understanding of the mechanisms behind reservoir persistence resulted in the development of several novel assays allowing to perform an extensive in-depth characterization. The objective of this review is to present an overview of the current state-of-the-art PCR-based technologies to study the replication-competent HIV-1 reservoir. Here, we outline the advantages, limitations, and clinical relevance of different approaches. Future HIV-1 eradication studies would benefit from information-rich, high-throughput assays as they provide a more efficient and standardized way of characterizing the persisting HIV-1 reservoir.

## 1. Introduction

The introduction of antiretroviral therapy (ART) has turned once deadly HIV-1 infection into a manageable chronic disease, although drawbacks include side effects, high costs, and requirement for lifelong adherence and accessibility to the medication [1]. However, a definitive cure is lacking, as ART interferes with the viral life cycle and halts viral replication but does not lead to HIV-1 eradication. Indeed, an interruption of ART typically results in viral rebound due to the early establishment of a so-called latent “HIV-1 viral reservoir”. This reservoir remains unaffected by ART and consists of a small stable pool of infected cells harboring HIV-1 proviruses that are replication-competent, forming the major barrier to a cure [2].

As the search for new HIV-1 cure strategies has been highly pursued over the last decade, it has put an emphasis on the need to adequately evaluate their efficacy by establishing an accurate way to quantify and genetically characterize the reservoir. Indeed, many ongoing clinical trials aim to reduce and/or eliminate the viral reservoir but lack a comprehensive and standardized assay to retrieve this type of information, making it often difficult to compare studies as the outcome depends on the assay that was used. 

Over the years, a wide range of different techniques have been developed to assess the persisting reservoir and can be stratified into two groups: 1) cell culture- or 2) PCR-based assays. Each of these assays has its merits and limitations, which need to be kept in mind in the setup of a study, as the desired information needs to be matched with the most relevant assay. 

The cell culture-based methods assess the activity and replication-competent status of the persistent reservoir via in vitro stimulation assays of HIV-1-infected cells (i.e., CD4+ T cells) and have been extensively discussed elsewhere [3,4]. Briefly, the main groups consist of viral outgrowth assays (VOA), the Tat/Rev induced limiting dilution assay (TILDA), and HIV-Flow methods enriching for infected cells expressing HIV-1 p24 protein after stimulation [5,6,7]. These methods unravel quantitative information on the replication competence of HIV-1 genomes but are tedious and often cumbersome to perform. In addition, they fail to reflect the real size of the HIV-1 replication-competent reservoir due to (i) the inability to reactivate all the latent virus with stimulation agents (underestimation) or (ii) the inability to distinguish protein or RNA products from defective and intact proviruses (overestimation) [3].

Next to this, a wide range of classical HIV-1 PCR-based assays exist, of which the total HIV-1 DNA and integrated HIV-1 assays are the most widely used in HIV-1 clinical trials. Unfortunately, these assays are not able to distinguish genetically intact and defective proviruses, as they focus on subgenomic regions, leading to gross overestimation of the replication-competent reservoir.

Overall, both groups of above-mentioned assays give information on different aspects on the HIV-1 reservoir but are not capable of accurately studying the replication-competent HIV-1 reservoir [8]. However, during the last years several new, improved PCR-based assays have been developed to obtain partial or complete sequencing information of the HIV-1 provirus. Hence, this review will capture these developments which are designed to further unravel the latent and replication-competent status of persisting HIV-1 reservoirs.

## 2. The Landscape of PCR-Based Methods to Asses HIV-1 Proviruses

**Figure 1:**
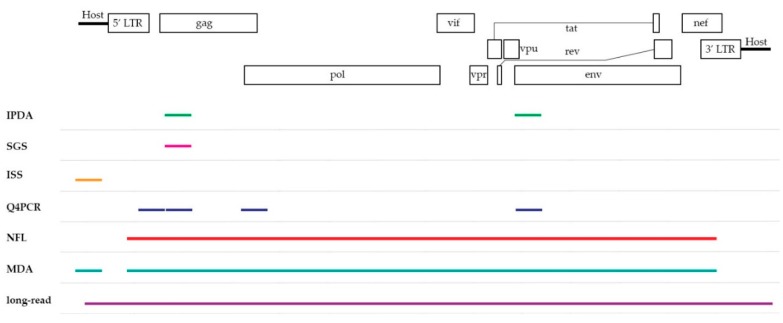
A schematic overview of the HIV-1 genome displaying the coverage of each assay discussed in this review. Note that the subgenomic coverage of SGS will depend on the target region of interest. IPDA = intact proviral DNA assay, SGS = single-genome/proviral sequencing, ISS = integration site sequencing, Q4PCR = quadruplex qPCR, NFL = near full-length sequencing, MDA = multiple-displacement amplification-based technologies, long-read = pooled CRISPR inverse PCR sequencing.

To overcome the major hurdle in current HIV-1 cure research, sensitive and accurate assays are needed to give an insight into the state of the viral reservoir and assist to determine the fraction of replication-competent HIV-1 proviruses. In the following section, a selection of PCR-based assays, listed in Table 1, will be discussed on their ability to define this viral landscape. They can be divided in two groups based on their coverage of the HIV-1 provirus as seen in Figure 1, yielding information on either subgenomic or the full-length provirus.

## 3. Subgenomic Methods

### 3.1. Intact Proviral DNA Assay (IPDA)

In an attempt to more accurately quantify the fraction of intact replication-competent HIV-1, Bruner et al. developed the IPDA on the two-color digital PCR QX200 system (BioRad) [9].

This assay consists of two duplex assays, one to detect HIV-1 and one for a human gene *RPP30*. The HIV-1 duplex assay combines information of two regions in the HIV-1 genome which are often deleted in replication-deficient viruses (packaging signal (PSI), *env*). Both these regions were selected based upon information on >900 full-length HIV-1 sequences and downstream bioinformatics analysis [9]. Next to deletions, the authors included a dark competition probe in the *env* assay to exclude viruses with hypermutations induced by APOBEC3G editing. Double-positive partitions are likely to contain an intact HIV-1 DNA virus. A control was implemented, indicating that the absence of probe signal was not wrongly interpreted as a HIV-1 deletion instead of a sheared genome during extraction protocol or due to handling of the samples. For this purpose, a duplex assay was designed for the *RPP30* gene with the same distance as for the HIV-1 duplex assay. As deletions are not expected for this human gene, single positives truly indicate shearing of DNA. Hence, a normalization can be performed on the HIV-1 assay based on this outcome, calculated as the DNA shearing index.

Bruner et al. demonstrated the capability of the assay in 62 patient samples. This method brings a medium-throughput assay for quantifying intact virus, which will be useful in the follow-up of HIV-1 cure clinical trials and more easily implemented as compared with full-length sequencing-based methods with more challenging protocols and bioinformatics analysis.

The IPDA remains a HIV-1 subtype B-oriented assay, so subtype-specific primers will be needed to quantify other subtypes. Indeed, an adaptation for the IPDA assay for SIV was recently described by the same group [21]. In addition, although this method prioritizes subgenomic regions for quantification of replication-competent HIV-1, it misses information on genomic variation elsewhere. Hence, it will still overestimate the fraction of intact viruses, but makes a much better estimation than the total HIV-1 DNA assays.

### 3.2. Single-Genome/Proviral Sequencing (SGS)

The single-genome/proviral sequencing techniques by Palmer et al. and Josefsson et al. presented the first opportunity to look at the genetic composition of the HIV-1 reservoir in plasma, cell subsets, or tissues by producing subgenomic DNA sequences from single HIV-1 viruses, ensured via a limiting dilution step [10,11]. Transitioning from sequencing at bulk to single copy level results in a more accurate overview, as viral quasispecies might be dominated by the major abundant proviruses [10]. The assay allows for a high-throughput study of the HIV-1 reservoir characteristics and dynamics including tropism determination, drug resistance mutations present in the target region, and phylogenetic analysis. As such, it has been used in several large-scale studies involving a wide range of samples to investigate the effects of new therapeutic compounds or identify the elusive source of viral rebound [22,23]. While the single-genome/proviral sequencing assay provides a comprehensive general overview of the persisting reservoir, it was designed to look at subgenomic regions of the HIV-1 virus such as the V1V3 *env* region and p6-RT *gag*-*pol* region. Thus, one should be aware that this SGS assay will report an overestimation of the number of replication-competent viruses, as one sequence might still be defective outside the genomic region that is not sequenced. Yet, due to the relative ease to perform SGS, it is still an effective way for phylogenetic analysis and to study small portions of the HIV-1 genome of interest, such as *pol* or *env*.

### 3.3. Integration Site Sequencing (ISS)

HIV-1 reservoir establishment is spearheaded by the stable integration of HIV-1 proviruses into the genomes of host cells, linking the fate of the provirus to that of the host cell. Indeed, the integration site plays a role in the activation state of the virus, where there is a fine balance between promoting latency and locking the virus in a dormant state. While it was long thought that reservoir persistence was the result of the long half-life of host cells or ongoing replication at sanctuary sites (or a combination of both), it has been shown that infected cells can undergo clonal expansion, as such contributing to the maintenance of the reservoir [12,13]. Clonal expansion of infected cells can be studied by two main techniques: proviral sequencing and integration site sequencing. While proviral sequencing provides qualitative data on reservoir composition, it is severely limited in studying clonal expansion by the assumption that identical proviral sequences stem from a clonally expanded infected cell. Indeed, when proviral sequence variability is limited, or when a small subgenomic region is sequenced, this assumption often does not hold true, as highlighted in a study by Laskey et al. [24]. ISS, however, does not have this drawback, as the recurrence of a specific integration site in a pool of infected cells is direct proof of clonal expansion. Methods for ISS can be broken down into two categories: ligation-mediated PCR followed by next-generation sequencing of bulk integration sites, and single proviral ISS after limiting dilution, which can be Sanger or Illumina sequencing based. The former type of method is based on shearing of DNA into small fragments, followed by ligations of adapters and PCR amplification of provirus/host DNA junctions [12]. The resulting amplicons are prepped and paired-end sequenced on an Illumina platform [12]. The fragmentation step introduces random breakpoints, which can be used to distinguish PCR duplicates from clonally expanded integration sites. The most important method falling into the latter category is the Integration Site Loop Amplification (ISLA) method by Wagner et al. [13]. This method is based on linear pre-amplification of proviral/host DNA junctions, followed by a looping step and nested PCR amplifications. The resulting amplicons are Sanger-sequenced. The main benefit of this method over Illumina-based methods is the fact that it allows to sequence a part of the provirus associated with a specific integration site, which is not the case for short-read Illumina-based methods. Therefore, most of the integration site sequencing techniques provide no information on proviral integrity, rendering them unable to focus on the replication-competent reservoir. 

However, the study of integration sites is contributing to our basic understanding of where HIV-1 integrates and which integration sites are persistent. Furthermore, enrichment of integration sites into genes that play a role in proliferation, combined with the observation of aberrant splicing events between the provirus and the host gene in vivo, highlighted the potential role of the integration site itself in driving clonal expansion [25,26]. The complex interplay between provirus and the neighboring host gene is a subject of great interest, as, to date, little is known in the context of HIV-1 persistence.

Nowadays, focus is shifting towards obtaining assays that can capture both the integration site and the proviral sequence, as this allows to study the relationship between replication competence and site of integration. Methods that can do both will be described later in this review.

## 4. Full-Length Methods

### 4.1. Near Full-Length (NFL) Sequencing

The first set of assays, like single-genome/proviral sequencing assays, provide no real estimate of the replication-competent viruses. Therefore, new assays have been developed to look at the intactness of individual proviral genomes, allowing for a good characterization of the genetic composition and thus making a better estimation of the replication-competent fraction of the persisting viral reservoir. In 2013, Ho et al. presented a novel technique to generate NFL proviral sequences [14]. Starting again with a limiting dilution to ensure the presence of a single proviruses per reaction, an outer PCR reaction was performed ranging from the 5′ LTR to the 3′ LTR. Next, inner PCRs targeting subgenomic regions were performed to verify hypermutations, followed by a set of inner PCRs amplifying overlapping regions of the HIV-1 genome of non-hypermutated HIV-1 positive wells. The resulting products were visualized on an agarose gel and cut out to send for direct Sanger sequencing followed by sequencing analysis. 

The initial results gave novel insights in the composition of the HIV-1 latent reservoir, revealing that the frequency of replication-competent proviruses was at least 60-fold higher than expected when measuring via a standard quantitative Virus Outgrowth Assay (qVOA), thus indicating the severe underestimation of this cell culture assay [14]. In 2016, a follow-up paper using the same technique by Bruner et al. studied proviruses present in non-induced CD4+ T cells from patients on ART [27]. A vast majority of proviruses were harboring a variety of genetic defects such as large internal deletions or APOBEC3G-mediated mutations. The observed fractions of intact proviruses were very low, 5% in patients who started ART during acute infection and 2% for patients starting ART during chronic infection. 

In 2017, two papers by Hiener et al. and Lee et al. presented a modified protocol, this time using a two-step nested PCR that amplifies a 9kb amplicon fragment (~92% of provirus), followed by deep next-generation sequencing (NGS) via Illumina [15,16]. For each positive well, de novo assembled proviruses using these short reads were generated and aligned to an HXB2 reference genome for further downstream analysis. Each provirus was assessed on a list of varying criteria such as inversions, large internal deletions, premature stop codons, frameshift mutations, and the presence of mutations or deletions in either the major splice donor (MSD) site or the PSI. This NGS strategy allows for a simplified, faster, and more cost-effective assessment of NFL proviral genomes than using multiple internal primers to sequence via Sanger, while also reducing the chance of erroneously labeling a virus as defective due to primer mismatch failing to amplify that region (however, it is important to note that the primers used in nested NFL PCR reactions might still show mismatches despite their location in a conserved region). 

These novel developments allow for an in-depth characterization of the viral reservoir, shedding a new light on the genomic composition and reservoir dynamics. Lee et al. studied several functionally polarized memory CD4+ T cells in five individuals of which Th1-polarized CD4+ T cells harbored the most intact [16]. Meanwhile, Hiener et al. looked at the proviral composition across different differentiation stages of CD4+ T cells in six participants, identifying effector memory T cells as the subset with highest amount of intact sequences [15]. Another study employing full-length NGS by Lu et al. sequenced proviral genomes from CD4+ T cells of 12 patients who underwent analytic treatment interruption (ATI) and compared those with results from VOA. They showed a partial overlap of intact sequences picked up from limiting dilution on lysed DNA from CD4+ T cells [28]. This demonstrates that the intactness of the sequence can be linked to replication competence, however no overlap with rebounding sequences was found [28]. Recent papers by Pinzone et al. and Lee et al. employed NFL sequencing to longitudinally study the evolution of the HIV-1 reservoir in different setups [26,29]. The former, spanning several years, focused on the selective pressures driving the dynamics of the viral reservoir in four individuals on ART and revealed a stronger clearance of intact proviruses due to a negative selection pressure driven by their effective protein expression [26]. The other paper by Lee et al. gave rare insights in the early stages of HIV-1 infection in four women infected with subtype C, observing the initial composition consisting of almost solely intact, non-identical proviral genomes and the increase in diversity of the proviral landscapes over time due to multiple factors such as truncation and hypermutation [29]. Additionally, all these studies report the detection of proportions of identical proviral sequences in several patients on ART, suggesting a role for clonal expansion of infected cells in the maintenance of HIV-1 reservoir. Yet, these NGS assays can merely lead to assumptions on clonality, as the integration site to confirm clonal origins is still missing [24].

The introduction of NFL sequencing provides a new level of understanding of the viral reservoir. Still, the costs associated with NGS due to use of limiting dilution are still high, and might explain why the aforementioned papers included, on average, six individuals, making more informative large-population studies too expensive [15,16,26,28,29].

### 4.2. Quadruplex qPCR (Q4PCR)

In 2019, Gaebler et al. presented a novel method using a combination of quantitative PCR (qPCR) and NGS to examine the latent HIV-1 reservoir dubbed Q4PCR [17]. 

This approach consists of a nested NFL PCR, but with an additional qPCR step between the outer and inner PCR reaction. The probes of their multiplex qPCR strategy cover four different regions of the HIV-1 region: packaging signal (PS), *gag*, *pol,* and *env*, which are optimized for optimal detection via in silico design using intact proviral sequences from the Los Alamos HIV Database. After the multiplex qPCR, only those reaction-positive for two probes are further selected for NFL PCR. Due to the inclusion of those four conserved regions, they can make a prediction on intactness. By adding this intermediate step, they can improve the efficiency of picking up intact or NFL proviruses by filtering out defective ones while still retaining qualitative data on non-sequenced wells (not observed on agarose gel comparison). The acquired intact sequences from six individuals were positive for any of two of the four probe combinations, as were 99% of the intact subtype B sequences on the Los Alamos HIV database, confirming the robustness of the assay design. 

Gaebler et al. present a sensitive, specific, and informative assay, capable of an unbiased characterization of the HIV-1 latent reservoir when compared with regular NFL NGS. It could help to reduce the costs associated with NGS of HIV-1 proviruses in larger clinical studies. Of note, the Q4PCR is not a standalone absolute quantitative assay as compared with the IPDA, as the quantitative nature of the Q4PCR requires a prior limiting dilution *gag* qPCR coupled with a near full-length PCR protocol.

### 4.3. Multiple-Displacement Amplification (MDA)-Based Techniques

As pointed out, clonal proliferation is thought to be a major driver of the viral persistence of the HIV-1 reservoir, as suggested by detection of similar integration sites via ISS and clusters of identical sequences via the current full-length assays. In 2019, Einkauf et al. and Patro et al. published similar novel approaches, respectively dubbed matched integration site and proviral sequencing (MIP-seq) and MDA-SGS, to get combined information indisputably linking proviral integrity and integration sites [18,19].

The setup is as follows: the first step includes an MDA at limiting dilution, which produces hundreds to thousands of long templates consisting of the proviral genome and the upstream and/or downstream host genomic sequence. Parts of these templates are subsequently used for viral sequencing by the SGS assay or NFL assay, and parts are used to go after the integration site with an ISS method of choice.

Both papers present the first combined data of proviruses and their chromosomal positions respectively on three and five participants. This offers a big change to earlier studies, which could only rely on assumptions of clonality based on expansion of identical viral sequences [24]. In addition to sequencing, Einkauf et al. also analyzed the chromatin accessibility and gene expression in proximity to integration sites to get a comprehensive overview of the persisting reservoir. On the basis of their observations, the researchers suggest the occurrence of deep latency, where patients on long-term ART would develop a reservoir consisting of less activatable, yet still intact proviruses due to their location in either non-genic/pseudogenic regions, their reverse orientation to the host gene, or location in less accessible chromatin [18]. Patro et al. used the new assay to explore clonality of proviral genomes in patients on ART, being either the result of clonal expansion of infected cells or the result of viral genetic bottlenecks during the absence of ART [19]. Like Einkauf et al., they noted the importance of reverse orientation to the host gene of integrated intact clones. Next, the authors were able to match an intact expanded clone to an NFL provirus picked up via VOA, confirming its replication-competent state.

The arrival of MDA-based techniques will allow for a better characterization of the HIV-1 reservoir. It allows the most informative view of the proviral landscape to date, providing insights in clonality and the replication-competent state of the persistent reservoir of individuals on ART and, more importantly, links these two together. Still, the associated high costs might hamper the wide use of this technique in large-scale studies.

## 5. Discussion and Future Perspectives

### 5.1. Shift from Partial to Complete Sequence Information

The ultimate goal is to obtain an assay capable of detecting the persisting HIV-1 reservoir and thus overcoming following technical challenges; (i) provide an accurate and sensitive overview of the replication-competent fraction while (ii) keeping the associated assay costs, duration, and complexity of the protocol at a minimum. Over the last decade, novel technological advancements have enabled the development and further fine-tuning of the discussed assays, with a shift from partial information to a more complete overview. So far, the MDA-based methods arise as the most informative and accurate techniques to date to measure the replication-competent HIV-1 reservoir, hereby overcoming the first technical challenge. Despite these assays claiming to be high-throughput, no data has been published of these assays being employed on large patient cohorts that could contribute to a more global level of understanding of HIV-1 persistence. An explanation can be found in the costs of these sequencing assays, since most protocols still resort to a limiting dilution step to ensure single viral templates and rely on the use of expensive reagents such as MDA enzymes, making these assays still very expensive to perform on very large cohorts. Next to this, the major focus of the studies so far has mostly been on CD4+ T cells derived from blood, a more in-depth look at other subsets and compartments that might contribute to the HIV-1 reservoir would also be advisable. No thorough comparison of these different approaches thus far has been carried out. In 2013, Eriksson et al. performed a cross-validating performance analysis for existing reservoir assays on a well-characterized patient cohort on ART. Redoing such analysis for the full-length sequencing assays benchmarked against the well-established assays would pinpoint the most efficient assay to use in future studies [8].

### 5.2. Pitfalls of PCR-Based Sequencing

Over the past decade, PCR and sequencing-based measures have gained increasing importance in HIV-1 research and cure studies. However, we should pay attention to pitfalls that are inherent to the mechanism of choice. HIV-1 is a virus with a high genetic variability between patients and within patients, which complicates optimizing an assay suitable for all HIV-1-patients since PCR is based on the identical match between an oligo and the target HIV-1 sequence, which is the foundation for all methods described here [30]. This inherent variation of the HIV-1 proviruses might affect the sensitivity and specificity of the assays, as they could still miss a “hidden” fraction of the reservoir due to mismatches [17,31]. In addition, most assays are currently solely optimized on HIV-1 subtype B, the most common subtype in the Western world, but not the most prevalent worldwide [32].

### 5.3. Need for Standardized Data Analysis

The emergence of these new sequencing-based technologies also results in new forms of data output and requires adequate bioinformatics pipelines to analyze the generated data while considering the complexity of HIV-1. The importance of this step should not be neglected, as different analysis strategies and different parameters could lead to different outcomes, making it hard to compare results between the assays. This gives rise to the need for good universal criteria on what defines replication competence, which factors should be taken into account while subjecting these sequences for analysis, and an open disclosure of the used strategy when presenting data. Examples of such pipelines offering a standardized method using Illumina sequencing data have been published, yet they focus on different aspects [33,34,35]. For instance, hivmmer is a variant-caller for the *pol* subgenomic region whereas shiver and veSEQ-HIV are both aimed at the reconstruction of HIV-1 quasispecies to study epidemiology and viral drug resistance [33,34,35]. So far, only two tools tailored for handling and analyzing individual NFL HIV-1 sequences have been published; HIVSeqinR by Lee et al. and Pro-Seq IT in Patro et al. [19,29]. Yet, a standardized analysis pipeline would benefit from a strict guideline on replication competence to avoid discrepancies between different users.

### 5.4. Promising Technology on the Horizon: Long-Read Sequencing

Next to the previously discussed assays that already have been used to generate clinically relevant data, another generation of assays is imminent, showing promise to overcome some limitations faced with the current assays, such as need for limiting dilution. Until now, all sequencing-based assays have made use of either Sanger or Illumina sequencing, resulting in short reads, restricted in size by technical limits of the method. Long-read and single-molecule sequencing, such as presented by PacBio and Nanopore, overcomes this size restriction and allows to sequence the whole provirus as a single read (and reducing the need for limiting dilution to guarantee single templates). Both technologies are maturing at a vast rate, with the associated error rate becoming less of an issue [36]. Exploration has been done in terms of applicability within the HIV-1 context, for example, by Artesi et al. [20]. In this preprint, the authors present a technique called pooled CRISPR inverse PCR sequencing (PCIP-seq) to asses retroviral integration and proviral genomes, mirroring the outcome of MDA-based techniques [20]. In short, genomic DNA is extracted, randomly sheared into fragments, and circularized, after which a CRISPR-guided enrichment strategy is employed to select for circles containing infected provirus. Afterwards, an inverse long-range PCR using HIV-1 specific primers is performed to produce amplicons. These fragments are sequenced via a long-read NGS technology, yielding reads that contain both provirus and part of the host integration site, allowing for an easier identification. In their preprint, the researchers present a proof-of-concept of this protocol on two HIV-1-infected patients on ART. They showcased the recovery of several integration sites like *STAT5B* (also found via ISS- and MDA-based techniques) and the (partial) genomes of the associated proviruses, enabling them to distinguish intact from defective viruses and detect the occurrence of clonally expanded cells [12,13,18,19,20]. Further optimization and increasing of sensitivity could lead to a novel approach while avoiding the use of limiting dilutions, which would make the protocol less laborious and expensive, thus offering a huge improvement on the second technical challenge.

### 5.5. Functional Confirmation of Replication-Competent HIV-1 and Beyond

While sequencing offers a way to look at the replication-competent state of the HIV-1 provirus, functional confirmation remains necessary to prove the studied provirus can be reactivated from its latent state. Therefore, linking intactness to actual replication competence is key, and assays that combine functional methods and sequencing would be highly informative, for instance, application of NFL sequencing after cell culture assays such as HIV-flow [7]. Still, other aspects of the viral reservoir besides proviral sequences and integration sites might also be of interest to study ongoing persistence. For example, several studies highlighted an important role for epigenetic modifications, like DNA methylation and histone modifications, affecting the transcriptional activity of the integrated proviruses [37,38,39,40,41]. Therefore, including assays like Hi-C, RNA-Seq, or assay for transposase-accessible chromatin using sequencing (ATAC-Seq) to study respectively the genomic confirmation and Hi-C contact densities near integration sites, the transcriptional activity of the host genes containing or surrounding integrated proviral HIV-1, and chromatin accessibility near integration sites would result into an even more comprehensive reservoir characterization [18,38,42]. The recent study by Einkauf et al. showcased this complementary approach employing both RNA-Seq and ATAC-Seq, as it allowed for a more in-depth discussion about the influence of proviral integration and its effect on latency due to the inclusion of chromosomal features [18]. Novel technologies like third-generation sequencing methods might lead to the development of integrated assays linking proviral information and chromosomal features as they allow for the identification of modified nucleotides, yet these techniques require untampered template DNA, since PCR would remove the epigenetic structure while applying bisulfite sequencing, or can result in short fragments unsuitable for long-read sequencing (i.e., ATAC-Seq) [18,39].

## 6. Closing Remarks

Over the past decade, analytical techniques have improved and brought us closer to determining the real size of the replication-competent reservoir. However, the development of a cost-effective, scalable approach for widespread use in clinical studies, which retains the level of information generated by methods like MDA-based sequencing methods, remains the ongoing challenge. This information is important as novel HIV-1 cure strategies are specifically targeting that small group of persisting infected cells, thus an accurate measurement is key to HIV-1 eradication. 

## Figures and Tables

**Table 1:** A selection of PCR-based assays. IPDA = intact proviral DNA assay, SGS = single-genome/proviral sequencing, ISS = integration site sequencing, Q4PCR = quadruplex qPCR, NFL = near full-length sequencing, MDA = multiple-displacement amplification-based technologies, long-read = pooled CRISPR inverse PCR sequencing.

	Assay	Assay Overview	Aspect	Advantage	Limitations	Key References
**Subgenomic coverage**	IPDA	ddPCR using two assays targeting subgenomic regions	Info on intactness and hypermutation	High-throughput Easy set-up Fast Quantification, better estimation than total HIV-1 DNA	Prone to overestimation No info on (full-length) sequence	[9]
	SGS	Sequencing at single-genome level	Info on intactness of subgenomic regions	Semi-high-throughput Single genome Easy set-up	Prone to overestimation No info on (full-length) sequence	[10,11]
	ISS	Sequencing of flanking host regions	Chromosomal Integration site	Detection of clonality Info on spatial context	No proviral sequence	[12,13]
**Full-length coverage**	NFL	Nested PCR, followed by Illumina-based sequencing at single-genome level	Full-length sequences	Relative high-throughput Distinction intact vs. defective provirus	More laborious workflow Cost No info on clonality	[14,15,16]
	Q4PCR	Modified NFL sequencing with addition of qPCR step for intactness filtering	Full-length sequences	See NFL Enrichment of full-length sequences	See NFL Increased bench time (qPCR)	[17]
	MDA	MDA at single-genome level, followed by NFL and ISS	Matching full-length and integration site	See NFL and ISS Combined info	Expensive Challenging workflow	[18,19]
	Long-read	Acquire single-genome and flanking host sequences via PCIP-seq in bulk	Fragments containing both full-length and integration site	See NFL and ISS Combined info Cost-effective compared to MDA	Lower sensitivity for small and/or non-clonal proviruses	[20]
